# HnRNP A1 controls a splicing regulatory circuit promoting mesenchymal-to-epithelial transition

**DOI:** 10.1093/nar/gkt579

**Published:** 2013-07-17

**Authors:** Serena Bonomi, Anna di Matteo, Emanuele Buratti, Daphne S. Cabianca, Francisco E. Baralle, Claudia Ghigna, Giuseppe Biamonti

**Affiliations:** ^1^Istituto di Genetica Molecolare, Consiglio Nazionale delle Ricerche (IGM-CNR), 27100 Pavia, Italy, ^2^International Centre for Genetic Engineering and Biotechnology, 34012 Trieste, Italy and ^3^Division of Regenerative Medicine, Stem Cells, and Gene Therapy, Dulbecco Telethon Institute at San Raffaele Scientiﬁc Institute, 20132 Milan, Italy

## Abstract

Epithelial-to-mesenchymal transition (EMT) is an embryonic program used by cancer cells to acquire invasive capabilities becoming metastatic. ΔRon, a constitutively active isoform of the Ron tyrosine kinase receptor, arises from skipping of *Ron* exon 11 and provided the first example of an alternative splicing variant causatively linked to the activation of tumor EMT. Splicing of exon 11 is controlled by two adjacent regulatory elements, a silencer and an enhancer of splicing located in exon 12. The alternative splicing factor and oncoprotein SRSF1 directly binds to the enhancer, induces the production of ΔRon and activates EMT leading to cell locomotion. Interestingly, we now find an important role for hnRNP A1 in controlling the activity of the *Ron* silencer. HnRNP A1 is able to antagonize the binding of SRSF1 and prevent exon skipping. Notably, hnRNP A1, by inhibiting the production of ΔRon, activates the reversal program, namely the mesenchymal-to-epithelial transition, which instead occurs at the final metastasis sites. Also, hnRNP A1 affects *Ron* splicing by regulating the expression level of hnRNP A2/B1, which similarly to SRSF1 can promote ΔRon production. These results shed light on how splicing regulation contributes to the tumor progression and provide potential targets to develop anticancer therapies.

## INTRODUCTION

Alternative splicing is a mechanism of gene expression regulation that either modulates the production of protein isoforms with distinct structural and functional properties or affects mRNA stability, through the introduction of premature stop codons, and translatability, by removing targets sites for microRNAs. Its prevalence in regulatory circuits is proven by the fact that >90% of human genes encode transcripts that undergo at least one alternative splicing event with a frequency higher that 10% (1,2). Moreover, alternative splicing contributes to the appropriate spatio-temporal regulation of cellular and developmental processes and to the response to a wide range of extracellular stimuli (3).

A detailed molecular analysis has revealed that alternative splicing decisions involve regulatory sequences, located both in exons and in the flanking introns, which promote (enhancers) or inhibit (silencers) the recognition of splice sites. These elements comprise the target sequences for RNA-binding proteins most of which belong to two groups of widely expressed antagonistic splicing regulatory factors: the SR (serine–arginine-rich) factors that usually promote exon recognition and the group of hnRNP (heterogeneous nuclear ribonucleoprotein) proteins, which in general play an inhibitory role (4). Notably, during tumor progression, stimuli from the tumor microenvironment may affect the expression and/or activity of splicing regulatory factors thus perturbing the physiological splicing program of genes involved in all major aspects of cancer cell biology, including cell cycle control, proliferation, differentiation, signal transduction pathways, cell death, angiogenesis, invasion, motility and metastasis (4–6). In many cases, unscheduled activation of splicing programs typical of embryonic development may occur. However, completely uncharacterized new splicing isoforms are frequently generated as well (7). An increasing body of evidence indicates that splicing variants of many cancer-related genes can directly contribute to the oncogenic phenotype and to the acquisition of resistance to therapeutic treatments (4–6). Hence, understanding the functional role(s) of cancer-associated alternative splicing variants and the mechanisms underlying their production offers the potential to develop novel diagnostic, prognostic and more specific anticancer therapies.

We have contributed to this topic by unveiling the connection between the expression level of splicing factor SRSF1 and the behavior of tumor cells (8). We have shown that SRSF1 (a member of the SR family also known as SF2/ASF) can regulate the epithelial-to-mesenchymal transition (EMT) and the migratory properties of cancer cells (8). EMT is a complex gene expression program through which terminally differentiated epithelial cells acquire mesenchymal features including the ability to efficiently move as single cells through the extracellular matrix (9,10). The EMT program is physiologically important during embryogenesis when it is crucial for organogenesis. However, in adults EMT occurs only during wound healing or it is involved in the metastatic spreading of epithelial cancers (9,10). SRSF1 is an oncoprotein upregulated in many human tumors (11). The involvement of SRSF1 in the EMT program derives from its ability to affect the splicing program of the tyrosine kinase receptor and proto-oncogene *Ron*. We have shown that SRSF1 promotes the production of ΔRon, a constitutively active isoform, through skipping of exon 11. More specifically, SRSF1 acts by directly binding to an exonic splicing enhancer (ESE) located in the constitutive exon 12 (8). As expected, additional factors such as hnRNP H and A2/B1 have a role in controlling splicing of *Ron* exon 11 and production of *ΔRo*n. Recently, hnRNP H has been shown to promote the expression of *ΔRo*n by binding to an exonic splicing silencer (ESS) in exon 11, while the mechanism of action of hnRNP A2/B1 has not yet been characterized (12,13). We have previously shown that the activity of the ESE bound by SRSF1 is counteracted by an ESS located upstream of ESE in the same *Ron* exon 12 (8). However, the molecular mechanism underlying the ability of the ESS to prevent skipping of exon 11 is still unknown.

In this manuscript, we report the characterization of the ESS element in exon 12 of the *Ron* gene. We show that the ESS is bound by hnRNP A1, a known antagonist of SRSF1 activity in splicing decisions (14). Intriguingly, binding of hnRNP A1 to the ESS sequence *in vitro* prevents the interaction of SRSF1 to the downstream ESE, which can be relevant for its ability to promote *Ron* exon 11 inclusion. In addition, hnRNP A1 impacts on the *Ron* splicing program by regulating the expression level of hnRNP A2/B1, which similarly to SRSF1 promotes *ΔRo*n production (13). Indeed, upregulation of hnRNP A1 induces alternative splicing in the 3′UTR of *hnRNP A2/B1* transcripts leading to mRNA degradation via the nonsense-mediated mRNA decay (AS-NMD) pathway (15). Consistently with its ability to inhibit the production of *ΔRo*n, hnRNP A1 activates the reversal of the EMT program, namely the mesenchymal-to-epithelial transition (MET). This activity may be crucial for the malignant process and for the formation of metastases because redifferentiation of mesenchymal cells to an epithelial state is required for the colonization of distant organs (9,10). Altogether, our analysis supports the hypothesis that antagonistic RNA-binding proteins may play an important role in the formation of metastases by modulating a specific splicing event tightly linked to the choice between EMT and MET programs. This will provide new insights toward the development of novel approaches to anticancer therapies.

## MATERIALS AND METHODS

### Cell culture, transfection, treatments and immunoﬂuorescence

Human embryonic kidney HEK-293 cells (American Type Culture Collection, CRL-1573) and human cervix carcinoma HeLa cells (ATCC, CCL-2) were grown in Dulbecco's modified Eagle's medium (DMEM) media supplemented with 10% fetal bovine serum (FBS) and 4 mM l-glutamine. Human gastric carcinoma KATOIII cells (ATCC, HTB-103) were grown in RPMI media supplemented with 20% FBS, 2 mM l-glutamine and 25 mM Hepes. The stable cell lines MDA-MB-435S (human breast cancer cell line, ATCC, HTB-129) expressing T7-SRSF1 or the empty vector pcDNA3.1(+) (Invitrogen) were grown in DMEM media supplemented with 10% FBS, 4 mM l-glutamine and 600 µg/ml G418.

We used Lipofectamine™ 2000 (Invitrogen) for transient transfection of HeLa, KATOIII and HEK-293 cells, Lipofectamine™ LTX and Plus Reagent (Invitrogen) for analysis of the endogenous *ΔRon* splicing in HeLa and MDA cells and Lipofectamine™ RNAiMAX (Invitrogen) for transfection of siRNA oligonucleotides, as recommended by the provider.

To study the sensitivity of splicing isoforms to NMD, HeLa and HEK-293 were treated with 10 µg/ml cycloheximide (Sigma-Aldrich) for 6 h before analysis.

Immunoﬂuorescence was performed on cells ﬁxed in 4% paraformaldehyde and stained with antibodies to anti-GFP (Roche) or anti-hnRNPA1 9H10 (Sigma-Aldrich) followed by indirect immunoﬂuorescence using FITC-conjugated anti-mouse antibody (Jackson ImmunoResearch Laboratories, Inc.). Nuclei were stained with 0.1 µg/ml DAPI (Sigma-Aldrich) and slides were then mounted with the MOWIOL reagent (EMD) and analyzed by using an optical microscope. Cell morphology of live cells was examined under a phase-contrast microscope (magnification 16×).

### Plasmids

*β-Globin* minigenes (H*β*-SIL-I, H*β*-SIL-II, H*β*-SIL-III and the mutated versions of H*β*-SIL-I) and *pRon* minigenes were generated by polymerase chain reaction (PCR)-mediated mutagenesis of H*β*-S and p2507-2991, respectively (8).

The GFP-tagged hnRNPA1 expression vector was provided by Prof. Claudio Sette (University of Rome Tor Vergata, Rome, Italy), whereas T7-hnRNPA1 was amplified by PCR from pCG-T7-hnRNPA1 (provided by Dr Javier Caceres, Medical Research Council Human Genetics Unit, Edinburgh, UK) and inserted into the EcoRI site of pcDNA3.1(+). All constructs were verified by sequencing.

### Reverse transcriptase-PCR

Three micrograms of DNase I-treated total RNA was retro-transcribed with d(T)_18_ oligo and Superscript II RT (Invitrogen). An aliquot (1/20^th^) was then PCR amplified. All primers are listed in Supplementary Table S1. Bands intensity on agarose gel was quantified with the NIH Image J program (version 1.44).

### Quantitative reverse transcriptase–PCR

For qPCR, an aliquot of the reverse transcriptase (RT) reaction was analyzed with QuantiTect SYBR Green PCR (QIAGEN) by using LyghtCycler 480 (Roche). Target transcripts levels were normalized to that of reference genes *GAPDH* or *hP0*. The expression of each gene was measured in triplicate in at least three independent experiments. All primers are listed in Supplementary Table S2.

### Western blot analysis

Cell were lysed in Laemmli buffer supplemented with complete mini EDTA-free protease inhibitor cocktail and phosphatase inhibitor cocktail PhosSTOP (Roche). Proteins were separated by sodium dodecyl sulphate–polyacrylamide gel electrophoresis and analyzed by western blotting by standard procedures (16).

The following primary antibodies were used: anti-hnRNPA1 9H10 (Sigma-Aldrich), anti-SRSF1 mAb96 (Invitrogen), anti-hnRNPH (Bethyl Laboratories), anti-hnRNPA2/B1 (Santa Cruz Biotechnology), anti-T7 mAb (Novagen), anti-GFP (Roche), anti-FLAG (Sigma-Aldrich) and anti-α-tubulin mAb (Sigma-Aldrich). Immunostained bands were detected by the chemiluminescent method (Pierce).

### *In vitro* RNA transcription and pull-down assay

RNA probes containing *Ron* splicing elements were transcribed according to established protocols (17), using as templates the minigenes pRon2507-2991 and pRon2507-2991-Mut-SIL I and the following set of oligos: T7_RonESS_for with RonSil_rev (SIL-I), T7_RonESS_for with RonESS_rev (*Ron* ESS), T7_RonESE_for with RonESE_rev (Ron ESE), T7_RonESS_for with RonESE_rev (*Ron* ESS + ESE). Sense and antisense oligos carried a T7 polymerase promoter sequence and a consensus-binding motif for TDP-43, respectively. *In vitro* pull-down assays were performed by standard procedures as described by Valacca *et al.* (18). Affinity purified proteins were analyzed by western blotting with anti-hnRNPA1 9H10 (Abcam), anti-SRSF6 (1H4, Invitrogen), anti-SRSF1 mAb96 (Invitrogen) and with rabbit polyclonal antibodies directed against TDP-43, PTB and hnRNP C (made in the F.E. Baralle laboratory according to standard immunization procedures).

### RNA immunoprecipitation

RNA-IP was carried out mainly as described in Cabianca *et al.* (19). Cells were seeded in 15 cm dishes (one for each RNA IP) and after 24 h were UV cross-linked with 100 000 μJ/cm^2^ twice (interval of 1′ between the two irradiations) on ice. Cells were then lysed in 0.5% NP40, 0.5% Na Deoxycholate, 300 U/ml Superase Inhibitor (Ambion), complete mini EDTA-free protease inhibitor cocktail (Roche) in phosphate buffered saline (PBS), pH 7.9, and put on rotation for 25′ at 4°C. Samples were treated with 30 U of Turbo DNase (Ambion) and incubated 15′ at 37°C. After centrifuging 5′ at 1350 g at 4°C, the supernatant was used in RNA-IP. For each RNA-IP, 100 μl of Dynabeads Protein G (Life Technologies) were conjugated with 10 μg of anti-hnRNPA1 9H10 antibody (Sigma-Aldrich) or IgG (Jackson ImmunoResearch Laboratories) as control. The antibody-conjugated beads were added to the samples (10% of the supernatant was saved as input) and put on rotation at 4°C over night. Beads were washed three times (5′ at 4°C) with PBS supplemented with 1% NP40, 0.5% Na Deoxycholate, additional 150 mM NaCl (final 300 mM) and 1:200 Superase Inhibitor (Ambion). Beads were resuspended in 100 μl of PBS + 10× DNase buffer and 3 U of Turbo DNase (Ambion). Samples were incubated 30′ at 37°C, with shaking. Beads were washed three times (5′ at 4°C) with PBS supplemented with 1% NP40, 0.5% Na Deoxycholate, 10 mM EDTA, additional 150 mM NaCl (final 300 mM) and 1:200 Superase Inhibitor (Ambion). RNA was eluted with 100 μl of 100 mM Tris HCl (pH 7.5), 50 mM NaCl, 10 Mm EDTA, 100 μg Proteinase K, 0.5% sodium dodecyl sulphate for 30′ at 55°C, with shaking. Eluate was centrifuged at 16 100*g* at RT and the supernatant was collected. RNA was purified with RNeasy Mini Kit (QIAGEN) according to the manufacturer’s instructions. After RNA resuspension in 30 μl of RNase-free water, samples were treated with Turbo DNase (Ambion). Five microliters of DNA-free RNAs were used in RT reactions with Superscript III supermix (Life technologies). All experiments were repeated at least three times. Quantitative PCR was performed on the following set of oligos: Ron_Exon12new_F and R, 7SL_F and R, 5 S_F and R, tRNALeu_F and R, A2B1_ 3′UTR_E and F (Supplementary Table S2).

### Wound healing assay

HeLa cells were transiently transfected with T7-hnRNPA1 or with the empty vector pcDNA3.1(+) as described above. The cells were grown in six-well plates to confluent monolayers and wounded after 24 h from transfection by dragging a 200 µl pipette tip through the monolayer. Cellular debris was removed by whasing with PBS (phosphate-buffered saline), and wound closure or cell migration images were monitored using an optical microscope (magnification 4×). Three fields of the wounded area for each dish were photographed at the initial wounding time (t0) and at the designed time points (5, 10 and 24 h after scratching). The open areas were measured at each time point by using the TScratch software (www.cse-lab.ethz.ch/software.html) (20). The migration rate was expressed as percentage of the wound closure over time, calculated by dividing the open area of postscratching times by the open area at t0. Three replicates from two independent experiments were performed.

## RESULTS

### Heterogeneous nuclear ribonucleoprotein A1 (hnRNP A1) interacts with the silencer in *Ron* exon 12

**Figure 1.**
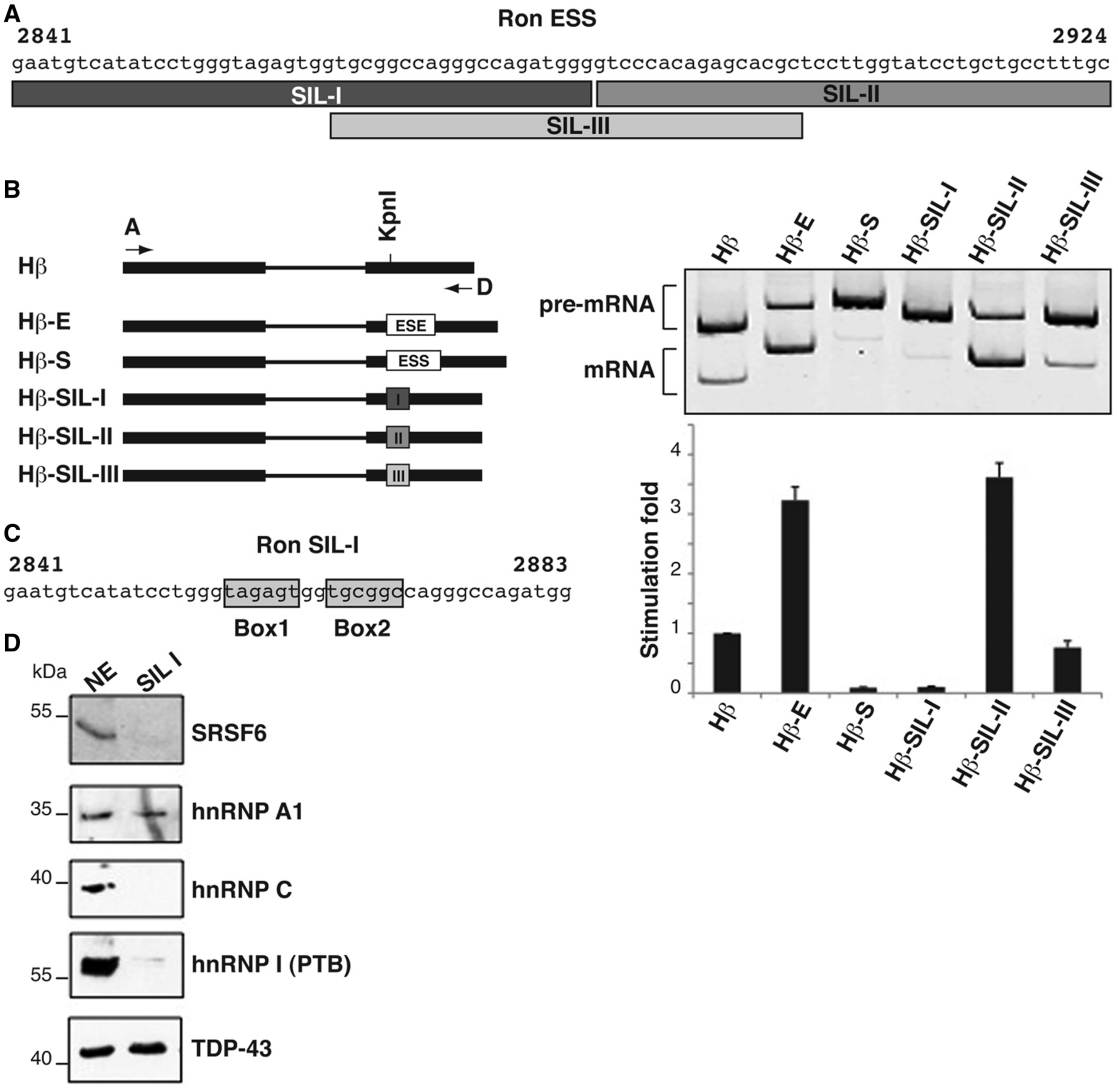
The first 44 nt of the *Ron* ESS element mediate splicing inhibition activity and interact with hnRNP A1. (**A**) Sequence of the previously identified 84 nt ESS. The position of SIL-I, SIL-II and SIL-III elements is indicated. Numbers refer to the cDNA sequence. (**B**) The entire ESS, SIL-I, SIL-II, SIL-III and ESE elements were cloned into the second exon of the human *β-globin* (Hβ) minigene. Minigenes were transfected into KATOIII cells; 24 h later RNAs were analyzed by RT-PCR with primer set β-globin_A and D (8) to detect both spliced (mRNA) and unspliced transcripts (pre-mRNA). Total (pre-mRNA plus mRNA, primers β-globin_A and Hbeta/CONTROL) and Spliced (mRNA, primers β-globin_A and Hbeta/DWsplicing) molecules were quantiﬁed by using qRT-PCR analysis; the ratio between Spliced and Total was plotted in the histogram by using the Hβ vector as a reference value. (**C**) Putative binding sites for hnRNP A1 (Box1) and SRSF6 (Box2) identified within SIL-I element by bioinformatics analysis. (**D**) *In vitro* pull-down assay with HeLa nuclear extracts (NE) and with a SIL-I riboprobe containing at its 3′ end the binding site for splicing factor TDP-43, as an internal control of pull-down efficiency. Materials were analyzed by western blotting with antibodies for indicated proteins.

We have previously reported the identification of two splicing regulatory elements, located in the central part (nt: 2841–2924) and at the 3′ end (nt: 2925–2991) of *Ron* exon 12 that correspond to an ESS and to an ESE, respectively (8). The activity of the enhancer is controlled by the splicing factor and oncoprotein SRSF1, which promotes skipping of exon 11, by directly binding to the *Ron* enhancer. We suggested that the primary function of the silencer element is to antagonize the activity of the ESE and to prevent exon skipping (8). To further characterize the silencer and to identify the interacting factor(s) that control its activity, we have applied an *in vivo* splicing assays in which different portions of the ESS element (84 nt) were cloned into the second exon of the human *β-globin* gene (Figure 1A and B) and tested for their ability to affect splicing of the upstream intron. As control, we used the *Ron* ESE element cloned in the same Hβ vector (Hβ-E plasmid) (8). Plasmids were transfected into gastric adenocarcinoma KATOIII cells in which preferentially skipping of endogenous *Ron* exon 11 takes place. After 24 h, RNAs were extracted and analyzed by RT-PCR with primers annealing to the *β-globin* sequences to detect both spliced and unspliced minigene transcripts. In agreement with our published results, the entire *Ron* ESS element (84 nt) drastically reduces splicing of *β-globin* transcripts (Hβ-S in Figure 1B). Surprisingly, while the first 44 nt of the ESS (SIL-I) are sufficient for efficient splicing inhibition, the last 40 nt of the ESS (SIL-II) behave as an ESE element and promote splicing of the minigene transcripts. A third region, SIL-III sequence (40 nt), which overlaps the 3′ and 5′ ends of SIL-I and SIL-II, respectively, does not show any effects with respect to the basal splicing profile of the empty Hβ construct (Figure 1B).

Based on this initial functional characterization, we focused on the SIL-I element. Bioinformatics analysis, performed with Human Splicing Finder (http://www.umd.be/HSF/) and SFmap (http://sfmap.technion.ac.il/) programs, identified within the SIL-I element (nt: 2857–2862) a putative binding motif (Box1 = TAGAGT in Figure 1C) for hnRNP A1. This box is similar to the hnRNP A1 ‘winner’ sequence [UAGGGA/U (21)] and identical to an intronic element by which hnRNP A1 modulates 5′ splice site selection of its own pre-mRNA (22). Moreover, the ESEfinder program (http://rulai.cshl.edu/cgi-bin/tools/ESE3/esefinder.cgi?process=home) identified a consensus binding motif for splicing factor SRSF6 (previously known as SRp55) between nt 2865 and 2870 within the *Ron* SIL-I element [USCGKM; S = G or C, K = U or G, M = A or C (23)] (Box2 = TGCGGC in Figure 1C). SRSF6 is a splicing factor of the SR family reported to interact with both enhancer and silencer sequences (24).

As a first step to understand whether hnRNP A1 and/or SRSF6 may control the activity of SIL-I element, we challenged the SIL-I sequence in an *in vitro* pull-down assay using a nuclear extract from HeLa cells. Interestingly, hnRNP A1, but not SRSF6, is able to interact with SIL-I riboprobe in this assay (Figure 1D), suggesting that the activity of *Ron* ESS element could be mediated by hnRNP A1.

### HnRNP A1 controls the activity of the *Ron* silencer

**Figure 2.**
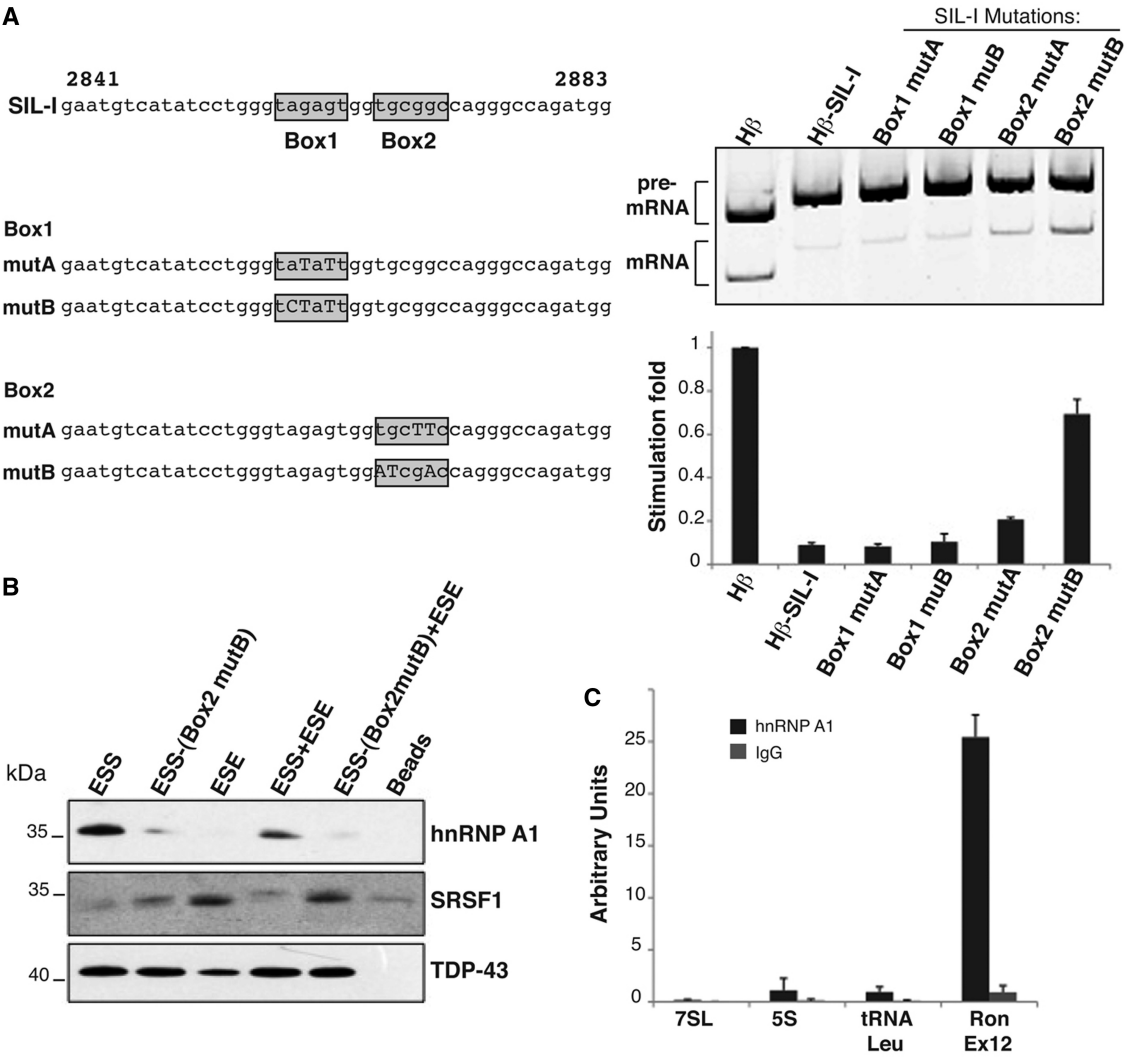
HnRNP A1 binding to the *Ron* ESS interferes with SRSF1 binding to the ESE element. (**A**) The panel on the left shows the sequence of the wild type SIL-I probe and its different mutated versions. Right panel: KATOIII cells were transfected with the indicated Hβ vectors and RNAs were analyzed by RT-PCR as in Figure 1B. (**B**) *In vitro* pull-down assay with HeLa nuclear extracts and the indicated RNA probes: WT *Ron* ESS (84 nt), ESS mutated in Box2 (mutB = TGCGGC replaced with ATCGAC), the *Ron* ESE (67 nt), ESE plus ESS elements (151 nt) WT or mutated in Box2. Affinity bound proteins were analyzed by western blotting with the indicated antibodies. (**C**) RNA immunoprecipitation (IP) following UV cross-linking using anti-hnRNP A1 mAb 9H10 or IgG on HeLa cells. The abundance of *Ron* exon 12 or, as control, 7SL, 5S and tRNA-Leu enrichments were measured by qRT-PCR.

HnRNP A1 is a well-established antagonist of SRSF1 (14). To investigate the relevance of hnRNP A1 binding for the activity of *Ron* ESS, we generated a series of Hβ-SIL-I minigenes bearing different mutations in the identified Box1 and 2 (Hβ-SIL-I-Mut in Figure 2A). Strikingly, the function of the silencer was not affected by mutations in Box1 that strongly matches the ‘winner’ sequence for hnRNP A1. On the contrary, mutations in Box2 (TGCGGC replaced with ATCGAC) strongly reduced the activity of SIL-I in KATOIII cells. Interestingly, Box2 is similar to the consensus binding motifs for the *Drosophila* homologs of hnRNP A1 (25). To understand whether hnRNP A1 binds to this sequence, we performed an *in vitro* pull-down assay using riboprobes containing the WT *Ron* ESS (84 nt) or an ESS mutated in Box2 (TGCGGC replaced with ATCGAC). As shown in Figure 2B, hnRNP A1 was selected by the wild type sequence but not by the riboprobe comprising the mutated Box2 (see lanes 1 and 2).

It has been reported that hnRNP A1, by interacting with RNA, can prevent the binding of SRSF1 to nearby sequences (14). Because Box2 is only 102 nt away from the SRSF1 binding site in exon 11, we decided to investigate whether or not the silencer could affect the interaction of SRSF1 with the ESE element. Therefore, we performed a pull-down assay using a riboprobe that comprises only the *Ron* ESE (cDNA 2925–2991) or a longer one (cDNA 2841–2991) that contains both the ESE and the ESS elements, either wild type or mutated in Box2. In accord with our previous data (8), SRSF1 associates with the ESE probe (Figure 2B, lane 3). However, no interaction of SRSF1 is detectable with the longer riboprobe containing the ESE plus the ESS (Figure 2B, lane 4). In keeping with our hypothesis, mutation of Box2 that prevents hnRNP A1 binding to the ESS rescues SFSF1 binding (Figure 2B, lane 5). These results suggest a possible competitive model in which hnRNP A1, by binding to ESS, can prevent the interaction of SRSF1 to the downstream ESE.

To verify that hnRNP A1 binds to the *Ron* ESS *in vivo*, we performed RNA immunoprecipitation following ultraviolet cross-linking (UV-RIP). Protein–RNA complexes were immunoprecipitated with anti-hnRNP A1 mAb or IgG, as a control. Immunoprecipitated RNAs were then analyzed by qRT-PCR. As shown in Figure 2C, *Ron* exon 12 was markedly enriched in the hnRNP A1 UV-RIP, whereas unrelated RNAs, such as 7SL, 5S rRNA and tRNA-leu, were not. As UV irradiation only identiﬁes direct protein–nucleic acid contacts (26), this result is supportive of a direct interaction of hnRNP A1 with *Ron* ESS *in vivo*.

Collectively, therefore, these results indicate that hnRNP A1 does bind to Box2 of the silencer in *Ron* exon 12 and that the interaction interferes with SRSF1 binding to the downstream ESE element.

### HnRNP A1 regulates splicing of the *Ron* gene transcripts

**Figure 3.**
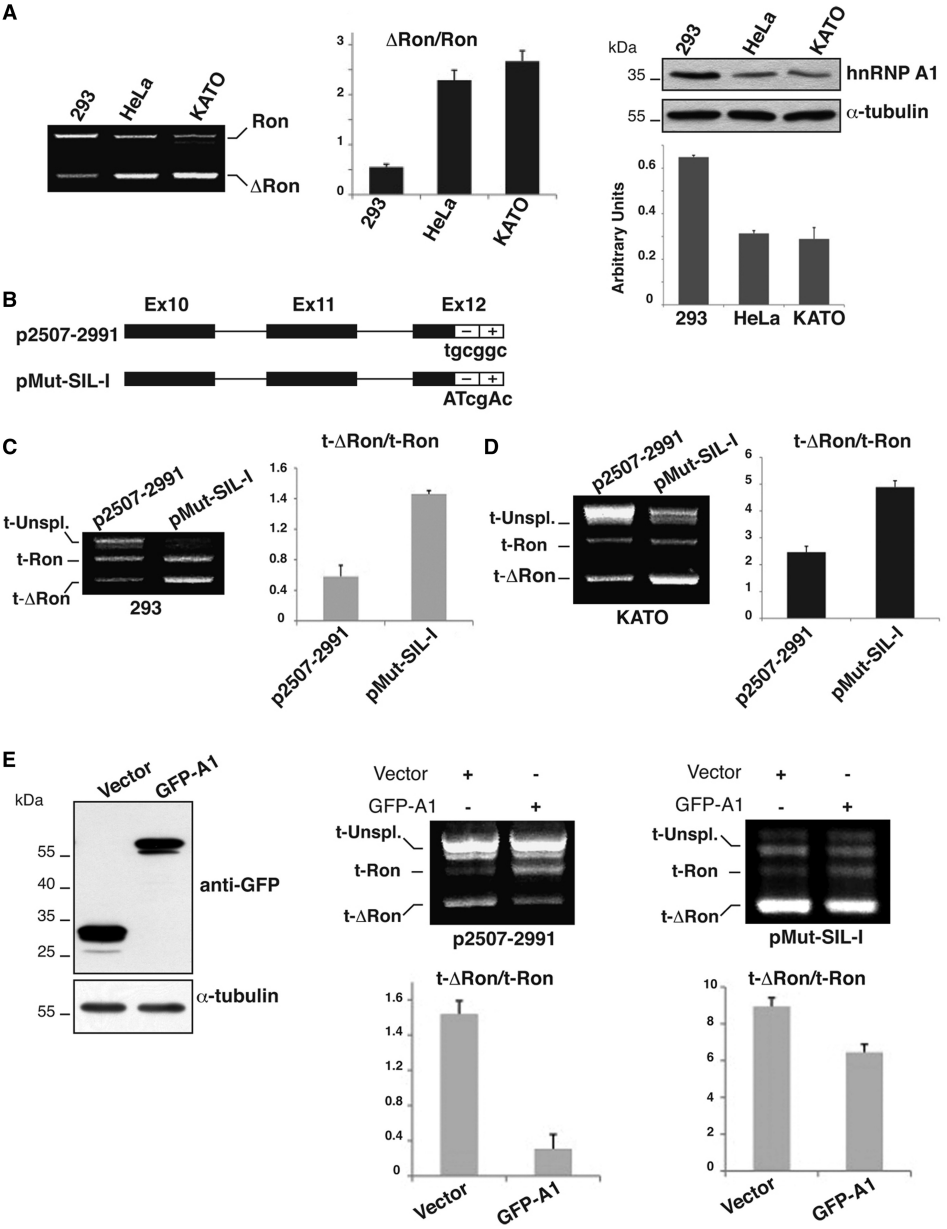
HnRNP A1 inhibits the production of the *ΔRon* transcript. (**A**) RT-PCR analysis of the splicing profile of endogenous *Ron* transcripts in HEK-293, HeLa and KATOIII cells with primers 2507 and 2991 (8). The histogram shows the qRT-PCR analysis of the total hnRNP A1 transcripts (primers hnRNPA1for and rev). On the right, western blot analysis of hnRNP A1 and α-tubulin protein levels in the indicated cell lines. (**B**) Scheme of the *Ron* minigene p2507–2991 (8) and of a derivative mutated in the ESS sequence (pMut-SIL-I). (**C**) HEK**-**293 and (**D**) KATOIII cells were transfected with the indicated minigenes and RNAs analyzed with primers 2507 and BGHrev (8); t-Ron and t-ΔRon indicate minigene transcripts generated by inclusion and skipping of exon 11, while histograms show the t-*ΔRon*/*t-Ron* measured in three independent experiments. (**E**) HeLa cells were transfected with GFP-hnRNP A1 or with the empty vector (‘Vector’). The expression of the overexpressed protein was verified by western blotting with anti-GFP and anti-α-tubulin antibodies. On the right, p2507–2991 and pMut-SIL-I minigenes were co-transfected into HeLa cells with the GFP-tagged hnRNP A1 or the empty vector. Total RNAs were analyzed by RT-PCR as in Figure 3B.

The results in the previous sections suggest a role for hnRNP A1 in preventing skipping of *Ron* exon 11. In agreement with this hypothesis, the expression levels of hnRNP A1 mRNA and protein in HEK-293, KATOIII and HeLa cells inversely correlates with the occurrence of *ΔRon* transcripts (Figure 3A). To verify the role of hnRNP A1 in controlling the splicing profile of *Ron* exon 11 *in vivo*, we compared the splicing pattern of transcripts encoded by the *Ron* minigene (p2507–2991 construct) (8), which contains exons 10, 11 and 12 along with the intronic sequences, with splicing of transcripts produced by a minigene mutated in Box2 (pMut-SIL-I) (Figure 3B). Minigenes were transfected into HEK-293 and KATOIII cells that respectively show high and low levels of hnRNP A1 (Figure 3C and D). Similarly to endogenous *Ron* transcripts, p2507–2991 minigene transcripts mainly show inclusion of exon 11 in HEK-293 and skipping in KATOIII cells (Figure 3C and D). In both cell lines mutation of Box2 reduces exon inclusion, indicating that the function of Box2 is relevant to control splicing of *Ron* transcripts regardless of the relative level of hnRNP A1.

To directly assess the effect of hnRNP A1 on the splicing profile of the *Ron* exon 11, either the wild-type or the Box2-mutated *Ron* minigenes were transfected into HeLa cells along with a plasmid directing the expression of a GFP-tagged hnRNP A1 (27) or with the empty vector, as control. As shown in Supplementary Figure S1, GFP-tagged hnRNP A1 properly localized in the nucleus with the same distribution pattern of the endogenous protein. HnRNP A1 over-expression inhibited skipping of *Ron* exon 11 resulting in a splicing profile similar to that observed in HEK-293 cells that contain a level of hnRNP A1 higher than HeLa cells (p2507–2991 in Figure 3E). Importantly, the mutation of the hnRNP A1 binding site in Box2 severely impaired the effect of hnRNP A1 over-expression (pMut-SIL-I in Figure 3E). These results strongly suggest an important role for hnRNP A1 in the regulatory circuit that controls the choice between inclusion and skipping of *Ron* exon 11.

### HnRNPA1 inhibits the production of ΔRon and promotes the MET

**Figure 4.**
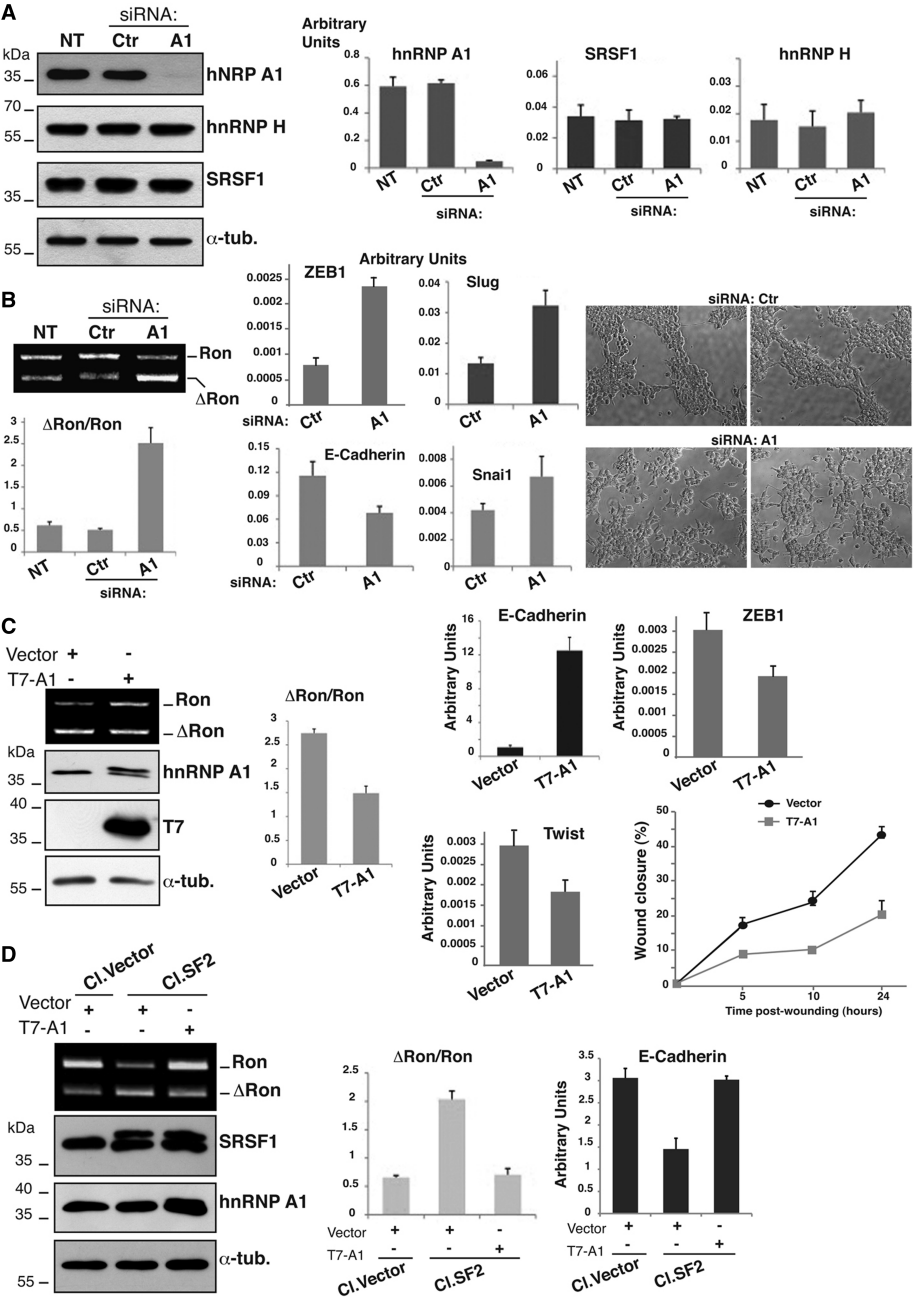
HnRNP A1 affects the MET program. (**A**) HEK-293 cells not treated (NT) or transfected with hnRNP A1 (A1) or with control siRNAs (Ctr). Total cell extracts were probed by western blotting with the following antibodies: anti hnRNP A1 (9H10), anti-hnRNP H, anti-SRSF1 (mAb96) and anti-α-tubulin; the three histograms on the right show the relative level of *hnRNP A1*, *SRSF1* and *hnRNP H* transcripts quantiﬁed by qRT-PCR. (**B**) The same cells (not treated or transfected with siRNA oligos) were analyzed in (i) RT-PCR to determine the splicing proﬁle of the endogenous *Ron* transcripts as in Figure 3A and (ii) qRT-PCR for the expression level of the indicated EMT markers. Cells morphologies were examined under a phase-contrast microscope (objective 10× with additional magniﬁcation 1.6×). (**C**) HeLa cells were transfected with T7-hnRNP A1 or with the empty vector (‘Vector’) with an efficiency of ∼60%; RNAs were analyzed with RT-PCR (as in Figure 3A) to determine the splicing profile of *Ron* exon 11 and cell extracts were analyzed in western blotting to assess the abundance of the indicated proteins and the expression of T7-hnRNP A1. The same cells were also analyzed in qRT-PCR for the expression levels of selected EMT markers. Panel on the right shows the results of the wound healing assay. Transient transfected HeLa cells were grown to confluence and wounded by dragging a 200 μl pipette tip through the monolayer (0 h); cell migration was measured at designated times (5, 10 and 24 h) after wounding. (**D**) MDA-MB-435S cells stably transfected with T7-SRSF1 (Cl.SF2) or with the empty vector (Cl.Vector) (29). Cl.SF2 and Cl.Vector cells were transfected with T7-hnRNP A1 or with the empty vector (‘Vector’) as indicated; total cell extracts were analyzed in western blotting to assess the level of the indicated proteins. (**E**) Total RNAs from the same cells were analyzed by RT-PCR (as in Figure 3A); the histogram below shows the *ΔRon*/*Ron* ratio. Expression level of the epithelial marker *E-cadherin* in the different cells as assessed by qRT-PCR is also shown.

Our results suggest that hnRNP A1 can inhibit the production of *ΔRon* transcripts by antagonizing the activity of the adjacent ESE element. As a first step to verify this hypothesis, we used previously validated siRNAs (28) to downregulate hnRNP A1 expression in HEK-293 cells that display a high level of hnRNP A1 and a low *ΔRon/Ron* mRNAs ratio (Figure 4A). This treatment did not affect the levels of SRSF1 and hnRNP H, another factor recently shown to control splicing of *Ron* exon 11 (12). As expected from our model, downregulation of hnRNP A1 was accompanied by skipping of *Ron* exon 11 and a higher *ΔRon*/*Ron* ratio (Figure 4A). We have previously shown that the level of ΔRon is a critical determinant of the transition from an epithelial to a mesenchymal cellular phenotype (EMT) (8). Thus, we asked whether down-regulation of hnRNP A1, by inducing the production of *ΔRon*, could trigger EMT. As shown in Figure 4B, down-regulation of hnRNP A1, in addition to stimulate *ΔRon* production, had evident morphological consequences. Upon transfection with siRNA against hnRNP A1 cells with long cytoplasmic protrusions and elongated, spindle-shaped morphology were detectable, whereas cells transfected with the control oligo were rounded and grew in clusters. This effect was accompanied by strong reduction of expression levels of the epithelial marker *E-cadherin* and increased expression of the mesenchymal markers *Slug, Snail1* and *ZEB1*, three repressors of *E-cadherin* (9,10).

The impact of hnRNP A1 on the EMT program is proven also by the reciprocal experiment in which we overexpressed hnRNP A1 in HeLa cells that displayed high fraction of *ΔRon* transcripts (Figure 3A). Interestingly, hnRNP A1 overexpression was sufficient to reduce skipping of *Ron* exon 11 (Figure 4C) and to induce upregulation of the epithelial marker *E-cadherin* accompanied by a decreased expression of transcriptional repressors, such as *Twist* and *ZEB1* (9,10) (Figure 4C). Importantly, in addition to prevent *ΔRon* splicing, hnRNP A1 overexpression reduced the migration properties of the cells as measured by a wound-healing assay (Figure 4C and Supplementary Figure S2). Altogether these experiments are consistent with a model whereby hnRNP A1, by antagonizing the production of *ΔRon* isoform, activates the reversal program of EMT, namely the MET, which occurs at the final metastatic sites and involves redifferentiation programs and conversion of mesenchymal cells to an epithelial state (9,10). Our model predicts that hnRNP A1 and SRSF1 have an antagonistic role in determining alternative splicing of *Ron* transcripts and thus in the choice between EMT and MET programs. To test this hypothesis we exploited a cell system based on human breast cancer MDA-MB-435S cells, which are characterized by low levels of *ΔRon* and SRSF1 (29). We have previously reported the generation of a clone of MDA-MB-435S cells (Cl.SF2) that stably overexpressed T7-SRSF1 at approximately the same level of the endogenous protein (Cl.SF2 in Figure 4D) (29). As expected, this clone shows a higher *ΔRon*/*Ron* ratio compared with MDA-435S cells stably transfected with the empty vector (Cl.Vector) (29). We wondered whether hnRNP A1 overexpression was able to revert the effect of SRSF1 overexpression in Cl.SF2. Transient hnRNP A1 overexpression was indeed able to antagonize SRSF1 and to reestablish the *ΔRon*/*Ron* ratio of the parental MDA-MB-435S (Figure 4D). Even more interestingly, hnRNP A1 overexpression drastically increases the level of the epithelial marker *E-cadherin* (Figure 4D). These results indicate that the antagonistic role of hnRNP A1 and SRSF1 in the regulation of *ΔRon* splicing results in an opposite effect of these two factors in controlling the EMT program and cell identity features. Moreover, the ability of hnRNP A1 to prevent *ΔRon* splicing appears to be a general phenomenon occurring in all the different cell lines tested so far.

### HnRNP A1 controls hnRNP A2/B1 expression through a highly conserved AS-NMD event

**Figure 5.**
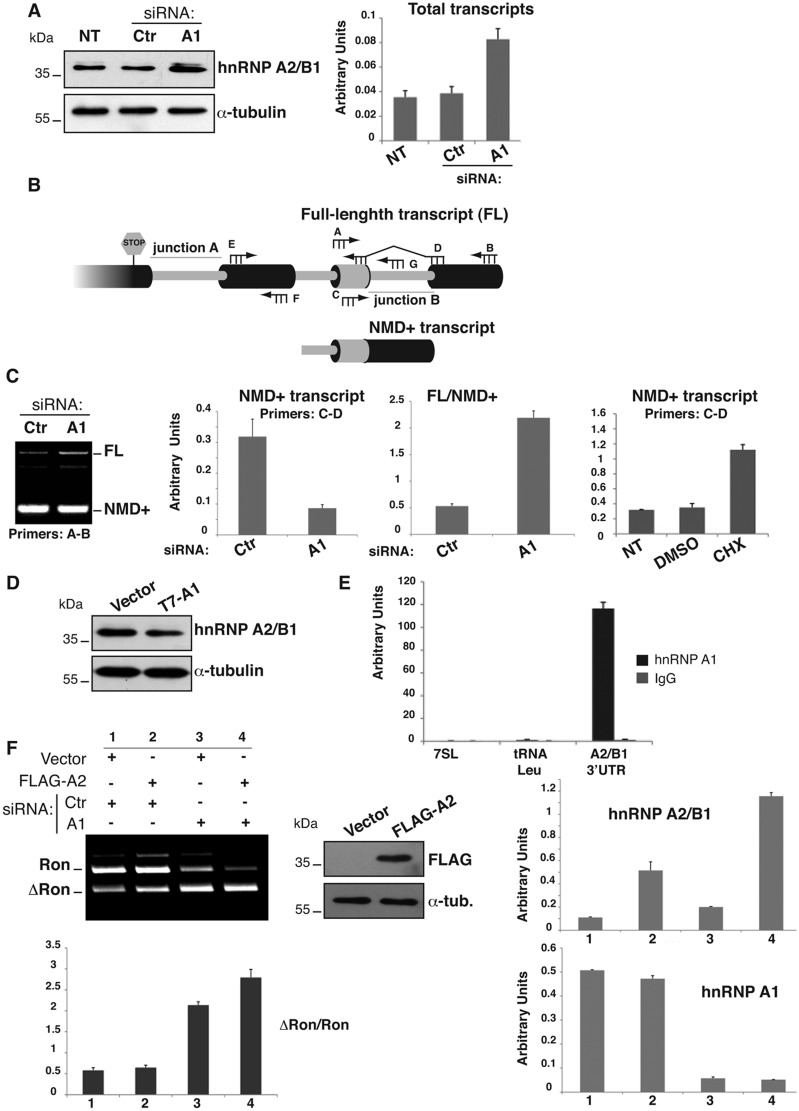
HnRNP A1 controls alternative splicing in the hnRNP A2/B1 3′UTR. (**A**) HEK-293 cells untreated (NT) or transfected with hnRNP A1 (A1) or with control siRNAs (Ctr) were analyzed by western blotting with anti-hnRNP A2/B1 and anti-α-tubulin antibodies and by qRT-PCR to determine the relative level of hnRNP A2/B1 transcripts. (**B**) Schematic representation of alternative splicing in the 3′UTR of *hnRNP A2/B1* transcripts. This cartoon represents the prediction of the exonic/intronic structure (boxes) by bioinformatics analysis (33). Intron retention generates the stable full-length (FL) transcript of *hnRNP A2/B1*, whereas splicing of the final intron in the 3′UTR (junction B) is expected to render all incumbent isoforms NMD sensitive (NMD+ transcript). The small intron (junction A) is too close to the Stop codon to transform it in a premature Stop codon on splicing. The position of primers used in RT-PCR analysis is also indicated. (**C**) Splicing proﬁle of *hnRNP A2/B1* transcripts (primers A2B1_3′UTR_A and B ﬂanking the last 3′UTR intron) in HEK-293 cells transfected with hnRNP A1 or control siRNAs. The histograms show (i) qRT-PCR analysis of the NMD+ transcript of *hnRNP A2/B1* (primers A2B1_ 3′UTR_C and D) in transfected HEK-293 cells, (ii) the ratio between the FL transcript (primers A2B1_ 3′UTR_C and G) and the NMD+ isoform of *hnRNP A2/B1* measured by qRT-PCR in the same treated HEK-293 cells and (iii) qRT-PCR analysis of the NMD+ transcript in HEK-293 cells not-treated (NT) or treated for 6 h with DMSO or CHX. (**D**) HeLa cells were transfected with T7-hnRNP A1 or with the empty vector (‘Vector’) and analyzed by western blotting to determine the hnRNP A2/B1 protein level. (**E**) RNA immunoprecipitation (IP) following UV cross-linking on HeLa cells. Protein–RNA complexes were immunoprecipitated with mAb 9H10 against hnRNP A1 or with control IgG. HnRNP A2/B1 3′UTR region (primers E and F) or, as control, 7SL, 5S and tRNA-Leu enrichments were measured by qRT-PCR as in Figure 2C. (**F**). HEK-293 cells were transfected with hnRNP A1 (A1) or with control (Ctr) siRNAs. After 24 h, the cells were transfected with flag-hnRNP A2 or with the empty vector (‘Vector’) and analyzed 24 h after a second round of siRNA treatment. The expression levels of hnRNP A1 and hnRNP A2/B1 were assayed both by qRT-PCR and by western blotting with antibodies anti-FLAG and anti-α-tubulin. The splicing proﬁle of the endogenous *Ron* transcripts was determined as in Figure 3A.

Three factors, SRSF1 (8), hnRNP H (12) and hnRNP A2/B1 (13), have been shown to promote skipping of *Ron* exon 11. We wondered whether hnRNP A1, in addition to modulate the splicing profile of *Ron* transcripts by direct binding to the ESS in exon 12, could also modulate the expression of the antagonistic factors. As shown in Figure 4A, after RNAi-mediated knockdown of hnRNP A1 we did not detect any variation in the level of SRSF1 and hnRNP H. Interestingly, however, we observed upregulation of hnRNP A2/B1 (Figure 5A), a factor known to regulate the EMT program and frequently overexpressed in cancer (13,30). It is known that, similarly to *SRSF1* transcripts (18,31,32), the *hnRNP A2/B1* pre-mRNA can undergo splicing of an intron in the 3′UTR region, leading to the production of a transcript (NMD+ transcript) degraded through the NMD (nonsense-mediated mRNA decay) pathway (33,34) (Figure 5B). Thus, we wondered whether the significant increment of hnRNP A2/B1 protein resulted from hnRNP A1 knockdown could be explained by hnRNP A1-mediated regulation of AS-NMD. As shown in Figure 5C, hnRNP A1 knockdown in HEK-293 cells is accompanied by an increase of the full-length (FL) *hnRNP A2/B1* mRNA and a contemporary decrease of the NMD sensitive isoform (NMD+). This is confirmed also by qRT-PCR quantification (Figure 5C). As predicted by the AS-NMD model, treatment of HEK-293 cells (Figure 5C) and HeLa cells (not shown) with cycloheximide (CHX), a well-known NMD inhibitor (15), produces a significant accumulation of NMD+ transcripts. In accord with this model, overexpression of hnRNP A1 in HeLa cells was sufficient to reduce hnRNP A2/B1 protein levels (Figure 5D). To understand if hnRNP A1 controls AS-NMD of *hnRNP A2/B1* by binding its 3′UTR region, we performed a bioinformatics analysis with the Human Splicing Finder program, identifying several putative hnRNP A1 binding sites in this region (not shown). Moreover, UV-RIP experiment shown in Figure 5E demonstrated that *hnRNP A2/B1* 3′UTR was highly enriched after hnRNP A1 immunoprecipitation, suggesting that hnRNP A2/B1 is a direct hnRNP A1 AS-NMD target.

Finally, to determine the relative contribution of hnRNP A2/B1 and hnRNP A1 proteins to the regulation of *Ron* splicing, we analyzed the splicing profile of endogenous *Ron* transcripts in HEK-293 cells overexpressing hnRNP A2/B1 and knockdown for hnRNP A1 (Figure 5F). Contrary to what previously reported by others (13), overexpression of hnRNP A2/B1 had no effect on Δ*Ron* splicing. Because HEK-293 cells are characterized by high levels of hnRNP A1, we hypothesized that hnRNP A2/B1 overexpression is not sufficient to counteract the effect of hnRNP A1. To test this hypothesis, we repeated hnRNP A2/B1 overexpression in the context of hnRNP A1 knockdown. Under these conditions, a modest, but reproducible, modification of splicing was detectable, with a 30% increase in exon 11 skipping (ΔRon). While these results are compatible with a role for hnRNP A2/B1 in the regulation of *Ron* splicing, they suggest that the contribution of hnRNP A2/B1 is relatively small at least in HEK-293 cells. It is plausible that, as predicted by the combinatorial model of splicing regulation, the relative contribution of different splicing factors may depend on the cellular context. Based on this, it is tempting to speculate that *Ron* splicing in HEK-293 cells is mainly controlled by antagonistic hnRNP A1 and SRSF1 factors.

## DISCUSSION

Deregulation of alternative splicing programs is a common feature of tumor cells (4,6) and contributes to the EMT, one of the major routes through which cancer cells acquire invasive capabilities and spread throughout the body as single cells (9). Changes of splicing profiles contribute also to the reversion of EMT, a process called MET, which is involved in the formation of metastasis and organ colonization (9). This EMT/MET cycle can be guided by stimuli in the tumor microenvironment and reflects the capacity of cancer cells to exploit gene expression programs that physiologically pertain to embryogenesis. By studying the *Ron* proto-oncogene, we provided the first example of an alternative splicing variant causatively linked to the activation of tumor EMT (8). Our studies opened the exciting possibility to consider splicing of *Ron* exon 11 as a target for the development of new anti-metastasis therapeutic approaches (29).

### Inclusion of *Ron* exon 11 is promoted by hnRNP A1

Three factors have been identified so far that promote skipping of Ron exon 11: hnRNP H that binds to an ESS within the exon (12), hnRNP A2/B1 whose mechanisms of action have not yet been clarified (13) and splicing factor SRSF1 that binds to an ESE at the 3′ end of downstream exon 12 (8). In addition to inducing the production of ΔRon, all these factors have also an effect on cell identity through activation of EMT program (30,35). Here we report the identification of the first splicing regulatory element and of its cognate splicing factor that promote inclusion of *Ron* exon 11. Paradoxically, the inclusion of exon 11 is promoted by a silencer of splicing, SIL-I, located in the 5′ half of exon 12. We suggest that the primary function of this element is to antagonize the activity of the downstream ESE bound by SRSF1. The activity of SIL-I depends on a sequence motif bound by hnRNP A1, a member of the hnRNP A/B subfamily with closely related sequence and a conserved modular structure (36). HnRNP A1 shuttles continuously between the nucleus and the cytoplasm and has fundamental roles in almost all major steps of mRNA metabolism such as constitutive and alternative splicing reactions (37), mRNA export (38), translation (39), microRNA biogenesis (40) and telomere maintenance. The expression level and activity of hnRNP A1 have been linked to different aspects of the tumor cell biology (36,41–44). Interestingly, hnRNP A1 is under the transcriptional control of the c-myc proto-oncogene and modulates the splicing of PKM2, activating the metabolic switch to aerobic glycolysis that is a hallmark of cancer cells (45).

HnRNP A1 controls splicing of exon 11 through a motif that does not match canonical consensus sites. However, noncanonical binding sites for hnRNP A1 have been already reported, for instance in *CEACAM1* exon 7 (43). Moreover, the unusual hnRNP A1 binding motif identified in *Ron* exon 12 resembles the consensus sequences for the *Drosophila melanogaster* homologs of mammalian hnRNP A1 (25), hrp36 (Hrb87F) and hrp38 (Hrb98DE), which share a high level of identity with human hnRNP A1 (respectively 51.9 and 54.2%) and the same structural organization (46). However, we cannot exclude that additional elements could contribute to the control of *Ron* splicing by hnRNP A1.

### Antagonistic activity of hnRNP A1 and SRSF1 in the choice between EMT and MET programs

In agreement with what was observed for other genes (47–49), also in the case of *Ron* gene, SRSF1 and hnRNP A1 exhibit an antagonistic role but with an effect on splicing opposite to that usually found, that is inhibiting and stimulating exon inclusion, respectively. How hnRNP A1 may exert its inhibitory activity is still a matter of investigation. As for the alternative splicing of the human immunodeficiency virus type 1 (HIV-1) *tat* exon 3 (50), it is conceivable that hnRNP A1 can form polymers able to prevent binding of additional splicing regulators to nearby sequences. A support to this hypothesis comes from an *in vitro* assay (Figure 2B), in which binding of hnRNP A1 to SIL-I prevents the interaction of SRSF1 with the downstream ESE.

A remarkable achievement of our analysis is that hnRNP A1, by antagonizing the production of ΔRon, activates redifferentiation MET process that in tumors is necessary for efficient metastasis colonization (51,52). Notably, hnRNP A1 regulates alternative splicing of other genes involved in invasion and metastasis (5). This is the case of the *Rac1* gene (41), where the constitutively active isoform *Rac1b* is preferentially produced in breast and colorectal cancers at various stages of neoplastic progression in response to extracellular stimuli (53,54). Rac1b is able to activate the EMT program in mammary epithelial cells on exposure to matrix metalloproteinase-3 (41). HnRNP A1 inhibits the production of Rac1b and, more importantly, downregulation of hnRNP A1 correlates with the progression from nonmalignant to malignant breast cancers (41). Downregulation of hnRNP A1 expression has been also observed in clear cell renal cell carcinoma (55), indicating that this is a feature frequently occurring during cancer development.

An increasing body of evidence indicates that splicing regulation, independently from transcription, can drive critical aspects of EMT-associated phenotypic changes (6). In particular, ESRPs and Rbfox2 factors have been recently linked to the acquisition of alternative splicing signatures of EMT and several of their targets, including genes encoding for proteins with well-known roles in organization of actin cytoskeleton, cell–cell adhesion, cell polarity and migration, have been identified (35,56–58). Whereas hnRNP A1 did not affect the expression levels of these splicing factors, we found that hnRNP A1 regulates the alternative splicing profile of two of their targets, such as *ENAH/MENA* and *SCRIB* (Supplementary Figure S3) (57,59). Similar to ESRPs and Rbfox2, hnRNP A1 knockdown increased skipping of *ENAH/MENA* exon 11, whereas increased exon 16 inclusion of *SCRIB* pre-mRNA (Panel B Supplementary Figure S3). Notably, splicing of exon 11A of *ENAH/MENA* gene has been shown to play a role in the EMT and to affect cellular motility and invasion (60,61). Notably, hnRNP A2/B1 overexpression did not affect the inclusion of exon 11A (13), suggesting that the effect of hnRNP A1 is not mediated by hnRNP A2/B1. Collectively, these findings indicate that a subset of the alternative splicing events from the global regulatory network of ESRPs and Rbfox2 is also co-regulated by hnRNP A1. Finally, our data provide new insights about the mechanisms through which hnRNP A1 could contribute to cellular transformation and cancer development.

### HnRNP A1 controls AS-NMD of *hnRNP A2/B1* transcripts

As stated above, two additional splicing regulators, hnRNP H and hnRNP A2/B1, share with SRSF1 the ability to promote the expression of ΔRon and EMT. Another one, Sam68, can be added to this list for its ability to modulate the level of SRSF1 via an AS-NMD event (18). The role of Sam68, thus, reveals the existence of a hierarchy through which splicing factors may control decisions relevant for EMT/MET programs. During our analysis we have found another example of hierarchic mode of regulation that involves the ability of hnRNP A1 to control AS-NMD of *hnRNPA2/B1* transcripts via splicing of an intron within the 3′UTR. As for SRSF1, AS-NMD of *hnRNP A2/B1* transcripts, in addition to provide a feedback mechanism for maintaining the homeostatic level of the protein (33), can be modulated by other splicing regulators in response to variations in growth conditions. The ability of the closely related hnRNP A1 to impact on AS-NMD of *hnRNP A2/B1* transcripts clearly reminds the PTB/nPTB system (62), another pair of closely related hnRNP proteins that reciprocally control their expression level through AS-NMD. However, differently from the PTB/nPTB example, hnRNP A1 can modulate *hnRNP A2/B1* mRNA level but not vice versa (13). The ability of hnRNP A1 to control hnRNP A2/B1 expression appears to be relevant for the biology of the cell because increased expression of hnRNP A1 in cancer cells has been shown to correlate with downregulation of hnRNP A2/B1 (42). Several observations indicate a prominent role of hnRNP A2/B1 in the alternative splicing of genes implicated in cellular migration and invasion (13,30,63). Indeed, upregulation of hnRNP A2/B1 correlates with poor prognosis in patients with glioma and its overexpression is sufficient to confer to mouse fibroblasts the ability to form high-grade sarcomas in nude mice (13).

## CONCLUSIONS

**Figure 6.**
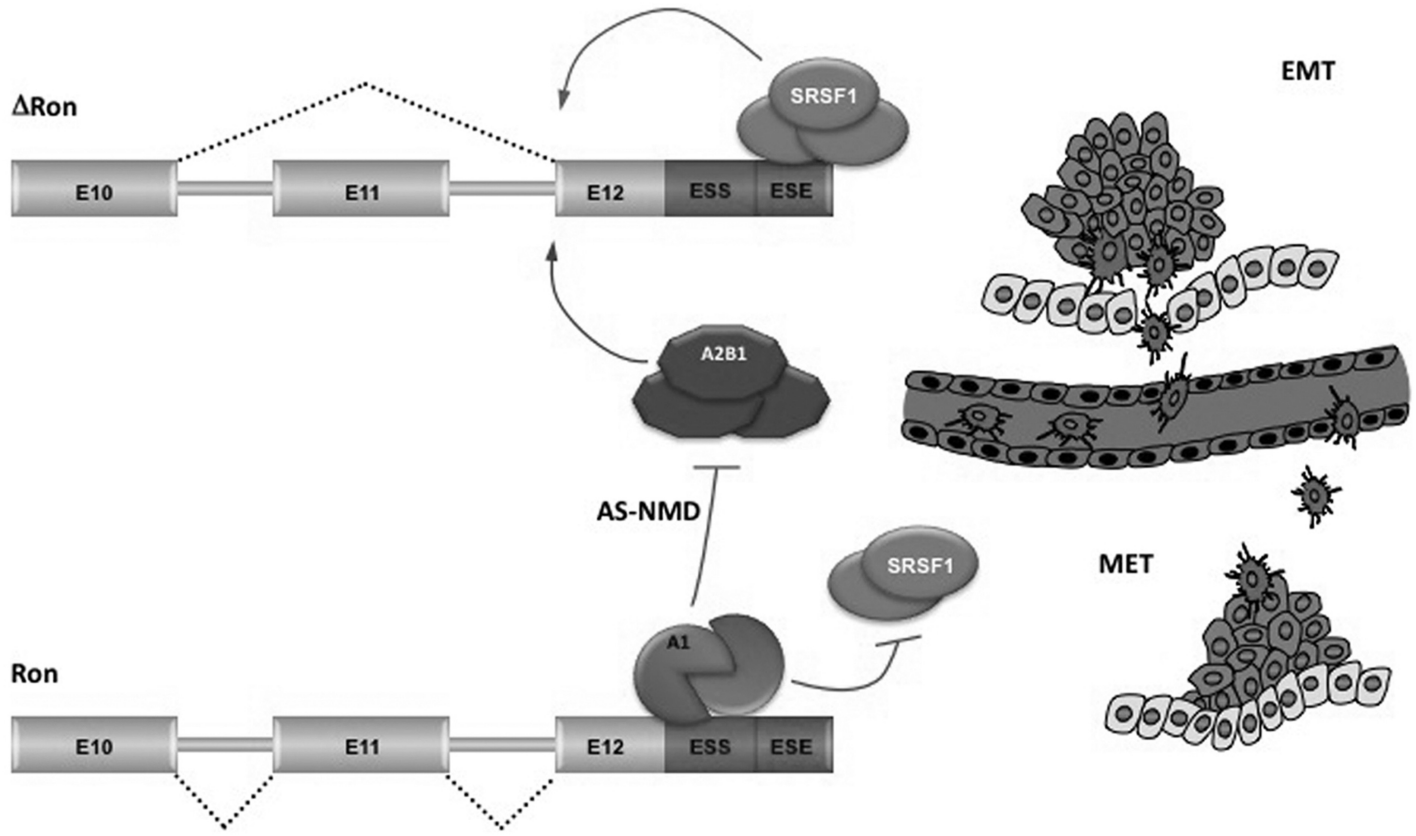
Different molecular mechanisms for the regulation of Δ*Ron* splicing during transient EMT-MET switches. Alternative splicing of *Ron* exon 11 depends on a ‘control cassette’ containing a silencer (ESS) and an enhancer (ESE) element, located in the constitutive exon 12. Splicing factor SRSF1 directly binds to ESE sequence, promotes the production of ΔRon isoform and triggers morphological and molecular changes typical of the EMT. In contrast, hnRNP A1, by binding to the ESS, counteracts association of SRSF1 factor to the adjacent ESE and promotes exon 11 inclusion. Thus, by inhibiting *ΔRon* production, hnRNP A1 prevents the EMT and sustains changes in the sense of a MET. HnRNP A1 also controls the expression levels of another ΔRon positive regulator (hnRNP A2/B1) through an AS-NMD event in the 3′UTR. The suggested implication of this splicing regulated circuit in tumor progression is illustrated by the scheme on the right. Cancer epithelial cells at the invasive front of the tumor undergo a dedifferentiation program through activation of EMT, which enables them to invade, intravasate and navigate through a network of thin vessels to initiate the formation of a secondary tumor. On reaching the metastasis sites, cancer mesenchymal cells revert back to their epithelial phenotype (MET) to allow growth and efficient metastasis colonization. A1: hnRNP A1; A2B1: hnRNP A2/B1.

Altogether, findings in this and in previous works support a molecular model for the regulatory circuits that control the definition (inclusion vs. skipping) of *Ron* exon 11. While SRSF1, hnRNP H and hnRNAP A2/B1 stimulate skipping of *Ron* exon 11, hnRNP A1 works at multiple levels to promote inclusion of this exon (Figure 6).

High levels of splicing factor SRSF1 that occurs in several epithelial cancers (11) promote skipping of the *Ron* exon 11 and increase the production of ΔRon. In this manner SRSF1 activates the EMT program, leading to cell locomotion (8). Upregulation of SRSF1 can be due to gene amplification (11,64), to c-myc-dependent increase of SRSF1 gene transcription (65) and, as we reported, to modulation of an AS-NMD event in the 3′UTR region of *SRSF1* transcripts (18). In contrast, high levels of hnRNP A1, by binding to the *Ron* silencer, counteract association of SRSF1 factor to the adjacent ESE and promote exon 11 inclusion. Thus, by inhibiting *ΔRon* production, hnRNP A1 prevents the EMT and activates the MET program. In addition to antagonize SRSF1 binding, hnRNP A1 also controls the expression levels of hnRNP A2/B1 through an AS-NMD event in the 3′UTR. The mechanism of action of hnRNP A2/B1 as positive regulator of ΔRon production remains to be elucidated.

## SUPPLEMENTARY DATA

Supplementary Data are available at NAR Online, including [66].

## FUNDING

Associazione Italiana per la Ricerca sul Cancro (to C.G. and G.B.); Progetto d’Interesse Invecchiamento (CNR-MIUR) (to G.B.); Association for International Cancer Research (AICR) (to C.G.). Funding for open access charge: Associazione Italiana per la Ricerca sul Cancro (to C.G. and G.B.).

*Conflict of interest statement*. None declared.

## Supplementary Material

Supplementary Data
