# Supplementary material for: HnRNP A1 controls a splicing regulatory circuit promoting mesenchymal-to-epithelial transition
Source: Nucleic Acids Res. 2013 Jul 17;41(18):8665–79. doi: 10.1093/nar/gkt579 (PMC3794575; doi:10.1093/nar/gkt579)
Supplement: Supplementary Data [file supp_41_18_8665__index.html]

HnRNP A1 controls a splicing regulatory circuit promoting mesenchymal-to-epithelial transition — HnRNP A1 controls a splicing regulatory circuit promoting mesenchymal-to-epithelial transition — Supplementary Data 

# HnRNP A1 controls a splicing regulatory circuit promoting mesenchymal-to-epithelial transition

## 

files

**Files in this Data Supplement:**

- Supplementary Data - jpg file
- Supplementary Data - jpg file
- Supplementary Data - jpg file
- Supplementary Data - doc file
