# Supplementary figures and images for: HnRNP A1 controls a splicing regulatory circuit promoting mesenchymal-to-epithelial transition
Source: Nucleic Acids Res. 2013 Jul 17;41(18):8665–79. doi: 10.1093/nar/gkt579 (PMC3794575; doi:10.1093/nar/gkt579)

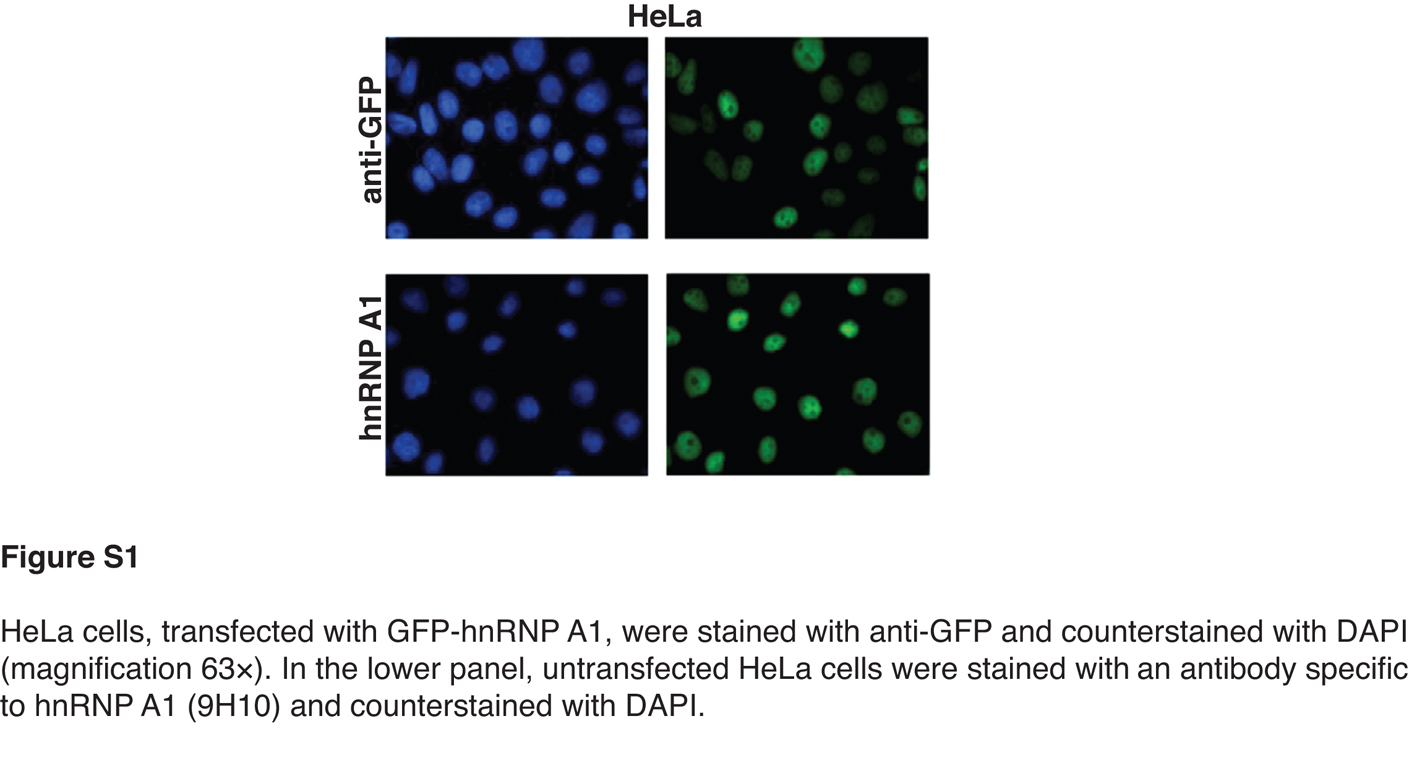

Supplement: Supplementary Data [file supp_gkt579_nar-00202-a-2013-File008.jpg]

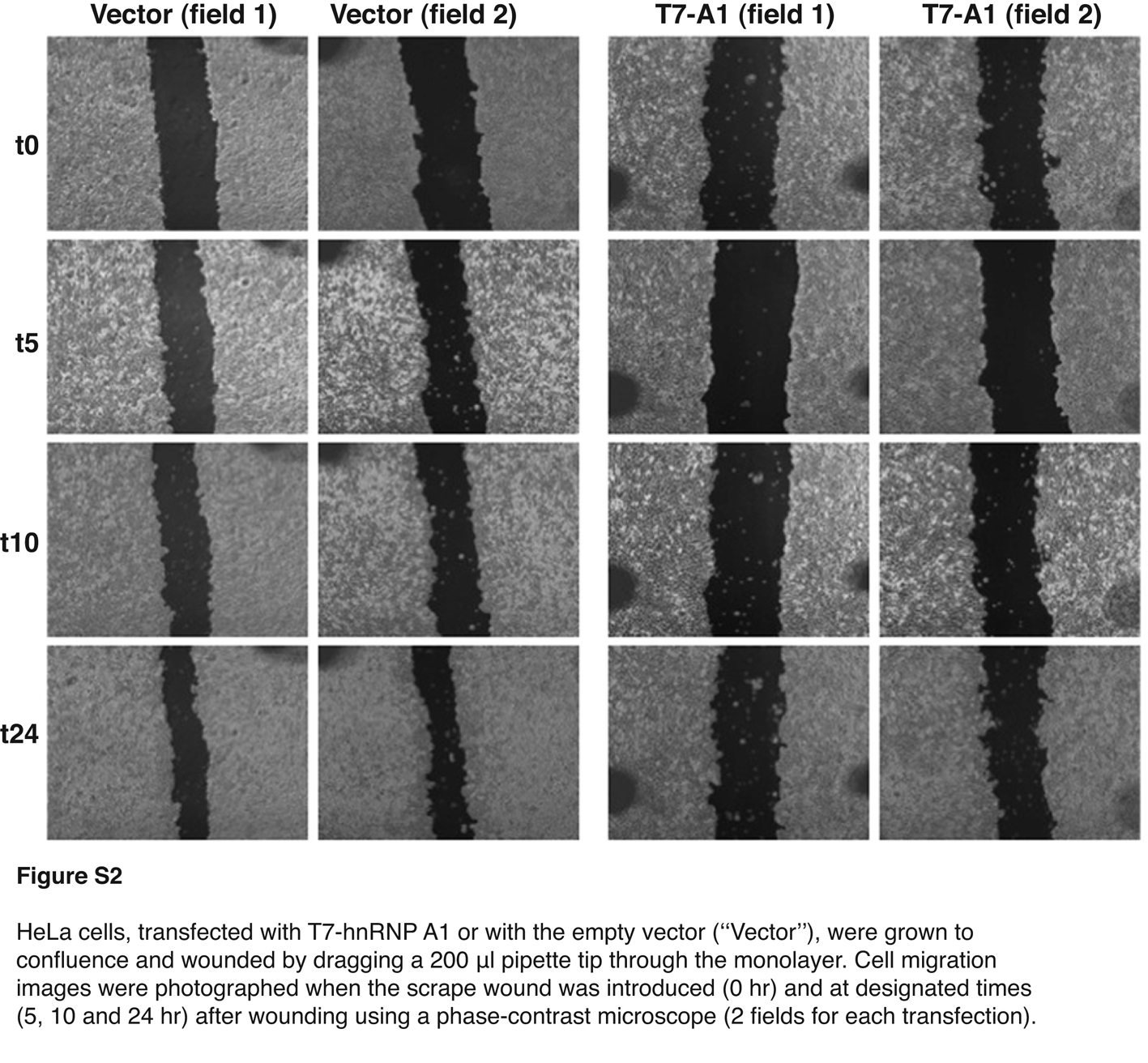

Supplement: Supplementary Data [file supp_gkt579_nar-00202-a-2013-File009.jpg]

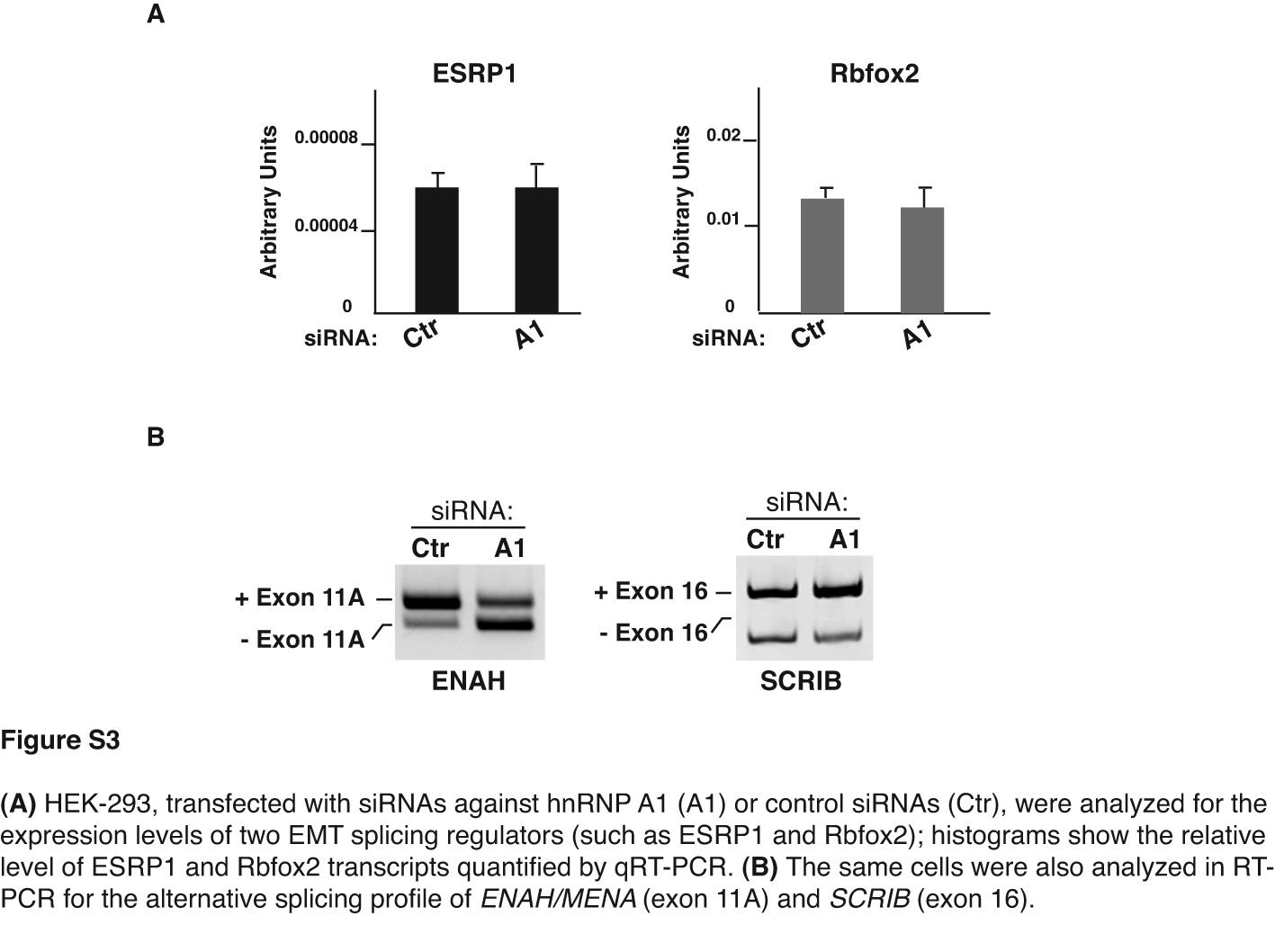

Supplement: Supplementary Data [file supp_gkt579_nar-00202-a-2013-File010.jpg]
