# Supplementary material for: HnRNP A1 controls a splicing regulatory circuit promoting mesenchymal-to-epithelial transition
Source: Nucleic Acids Res. 2013 Jul 17;41(18):8665–79. doi: 10.1093/nar/gkt579 (PMC3794575; doi:10.1093/nar/gkt579)
Supplement: Supplementary Data [file supp_gkt579_nar-00202-a-2013-File011.doc]

Table S1. **Primers used in PCR experiments**

| **Primer** | **Sequence (**5’--3’) |
| --- | --- |
| A2B1_3’UTR_A | TTTCAACAGCTGAGGCAAGC-3’ |
| A2B1_3’UTR_B | AGTTACCTGCAGCAAGACACC |
| T7_RonESS_for | TAATACGACTCACTATAGGGAATGTCATATCCT |
| RonSil_rev | CACACACACACACACACCATCTGGCCCTGG |
| RonESS_rev | CACACACACACACACAGCAAAGGCAGCAGGA |
| T7_RonESE_for | TAATACGACTCACTATAGGTGCTGCTTGTGGCT |
| RonESE_rev | CACACACACACACACACTAGCTGCTTCCTC |

Table S2. **Primers used in qPCR experiments**

| **Primer** | **Sequence (**5’--3’) | **Reference** |
| --- | --- | --- |
| HPO/F | ATGCCCAGGGAAGACAGGGCG |  |
| HPO/R | CGAAGGGACATGCGGATCTGCTGC |  |
| GAPDH-1_for | TCTCCTCTGACTTCAACAGCGACA |  |
| GAPDH-1_rev | GACAAAGTGGTCGTTGAGGGCAAT |  |
| Hbeta/CONTROL | TGCCCAGGGCCTCACCACCAACTTC |  |
| Hbeta/DWsplicing | CACCAGCAGCCTGCCCAGG |  |
| hnRNPA1for | ACAACTTCGGTCGTGGAGGAAACT |  |
| hnRNPA1rev | CCAAAATTGCTTCCATCATTACCAA |  |
| hnRNP A2/B1_for | GCTGTAGCAAGAGAGGAATCTGGA |  |
| hnRNP A2/B1_rev | GCTTCTTCACAGTTACATGAGCCC |  |
| A2B1_ 3’UTR_C | TCTGCTGCCACAAAGACTGTA |  |
| A2B1_ 3’UTR_D | GCAGCAAGACACCTTCCATT |  |
| A2B1_ 3’UTR_E | ATGCTGCTTCTTCAATGCAA |  |
| A2B1_ 3’UTR_F | AGCTTTCGTTGGCATCCTTA |  |
| A2B1_3’UTR_G | AAGGGTGTAACGATGGGAAA |  |
| hnRNPHfor | GAAGTCAAATTGGCCCTGAA |  |
| hnRNPHrev | CCCCAGGTCTGTCATAAGGA |  |
| SRSF1_for | CTCCAAGTGGAAGTTGGCAGGATT |  |
| SRSF1_rev | ACACCAGTGCCATCTCGGTAAACA |  |
| Rbfox2_F | CAGACCGGTGAGCATAACCT |  |
| Rbfox2_R | TAGAGACATGCAGCCGTTTC |  |
| Esrp1_F  Esrp1_R | CAGAGGCACAAACATCACAT  AGAAACTGGGCTACCTCATTGG | 1 |
| ENAH_F | CAAGAAAACCTTGGGAAAGAA |  |
| ENAH_R | CAATGAGCTCTTCTTTTAGCTTTG |  |
| SCRIB_F  SCRIB_R | GACAAGGAGGGGGCCGTGGTTTCT  TATGCCCTCGTCGTCCCCCTTAT | 2 |
| Ecad_H_for | CTGGGACTCCACCTACAGAAAGTT |  |
| Ecad_H_rev | GAGGAGTTGGGAAATGTGAGCA |  |
| Slug_H_for | ACCTTGTGTTTGCAAGATCTGCGG |  |
| Slug_H_rev | TGCAAATGCTCTGTTGCAGTGAGG |  |
| Snail (ex2) dir | TTGGAGGCCGAGGCCTATGC |  |
| Snail (ex2-3) rev | GGCTTCTCGCCAGTGTGGGT |  |
| Twist_F | GTCCGCAGTCTTACGAGGAG |  |
| Twist_R | CCAGCTTGAGGGTCTGAATC |  |
| ZEB1_F  ZEB1_R | AAGAATTCACAGTGGAGAGAAGCCA  CGTTTCTTGCAGTTTGGGCATT | 3 |
| Ron_Exon12new_F | TCTGCGTAGATGGTGAATGTC |  |
| Ron_Exon12new_R | GCCACCAGTAGCTGAAGACC |  |
| 7SL_F | GTGTCCGCACTAAGTTCGG |  |
| 7SL_R | TATTCACAGGCGCGATCC |  |
| 5S_F | TACGGCCATACCACCCTGAA |  |
| 5S_R | GCGGTCTCCCATCCAAGTAC |  |
| tRNALeu_F | GTCAGGATGGCCGAGTGGTCTAAG |  |
| tRNALeu_R | CCACGCCTCCATACGGAGACCAGAAGACCC |  |

**References:**

**1.** Warzecha,C.C., Sato,T.K., Nabet,B., Hogenesch,J.B. and Carstens,R.P. (2009) ESRP1 and ESRP2 are epithelial cell-type-specific regulators of FGFR2 splicing. *Mol. Cell*, **13**, 591-601.

**2.** Warzecha,C.C., Jiang,P., Amirikian,K., Dittmar,K.A., Lu,H., Shen,S., Guo,W., Xing,Y. and Carstens,R.P. (2010) An ESRP-regulated splicing programme is abrogated during the epithelial-mesenchymal transition. *EMBO J.*, **6**, 3286-3300.

**3.** Wellner,U., Schubert,J., Burk,U.C., Schmalhofer,O., Zhu,F., Sonntag,A., Waldvogel,B., Vannier,C., Darling,D., zur Hausen,A., et al. (2009) The EMT-activator ZEB1 promotes tumorigenicity by repressing stemness-inhibiting microRNAs. *Nat. Cell. Biol.*, **11**, 1487-1495.
